# P-2204. Human Parainfluenza Virus 1–4 Illnesses in the HIVE Cohort During 2010–2022

**DOI:** 10.1093/ofid/ofaf695.2367

**Published:** 2026-01-11

**Authors:** Joshua E Foster-Tucker, Arnold Monto, Amy Callear, Rachel Truscon, Matthew R Smith, Elie-Tino Godonou, Emily T Martin

**Affiliations:** University of Michigan School of Public Health, Ann Arbor, MI; University of Michigan, Ann Arbor, MI; University of Michigan School of Public Health, Ann Arbor, MI; University of Michigan School of Public Health, Ann Arbor, MI; University of Michigan, Ann Arbor, MI; University of Michigan School of Public Health, Ann Arbor, MI; University of Michigan, Ann Arbor, MI

## Abstract

**Background:**

Human parainfluenza viruses (HPIVs) cause significant annual acute respiratory illness (ARI) burdens in the U.S. Yet, HPIV circulation and ARI characteristics are not fully understood, especially for HPIV-4, which may be an underrecognized cause of moderate and severe ARI. We describe the circulation and ARI features of HPIVs 1–4 in the Household Influenza Vaccine Evaluation (HIVE) cohort over 12 years.

**Figure:**
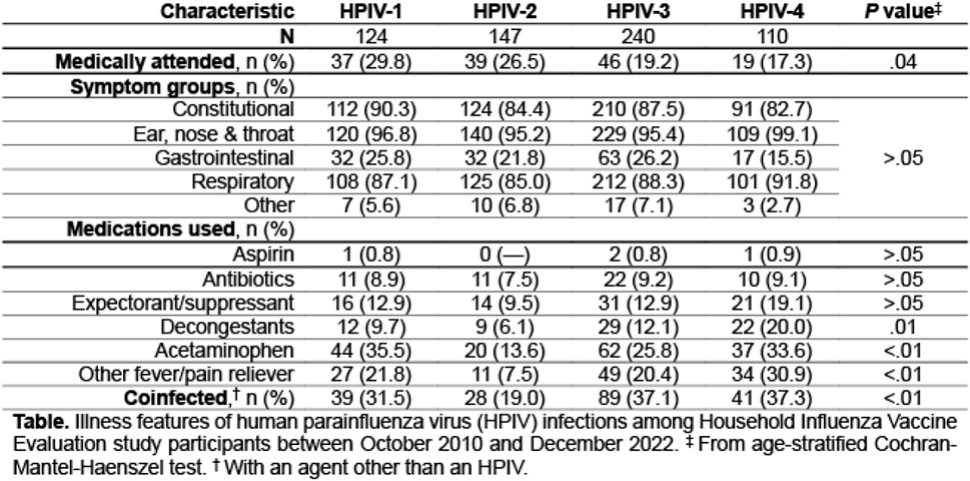

**Methods:**

The HIVE study has followed a household cohort in Ann Arbor, MI, since fall 2010, with active study during fall–winter influenza seasons until July 2015 and year-round thereafter. HPIV ARIs may have been under-detected before July 2015, especially HPIV-3, which peaks in summer. This analysis used HIVE data from October 2010 to December 2022. Participants provided swabs for ARIs meeting our case definition of ≥2 ARI symptoms (e.g., cough & fever) with onset during the prior week, which were tested for HPIVs 1–4. Ill participants reported symptoms, medication use, and healthcare sought, with statistical comparisons adjusted for onset age.

**Results:**

During follow-up, 13758 ARIs were reported, with 616 (4.5%) positive for ≥1 HPIV. HPIV-3 was identified in 39% of swabs, HPIV-2 in 23.9%, HPIV-1 in 20.1%, and HPIV-4 in 17.9%. Median ages at HPIV-1 (5.6 y) and -3 (5.8 y) ARI were younger than HPIV-2 (7.3 y) and -4 ARI (7.6 y). Children under age 12 accounted for 69.1% of all HPIV ARIs. Infection frequencies by sex and race were similar. Pre-pandemic circulation aligned with established seasonal patterns; all four HPIVs resumed circulation by fall 2022. The Table shows significant variability in medically attended ARI frequency across HPIVs (range, 17.3% HPIV-4 to 29.8% HPIV-1; *p* = .04). Medication use during HPIV-4 ARI was frequent. Significant variations in decongestant, acetaminophen, & other fever/pain reliever usage and coinfection occurrence were noted across HPIVs (all *p* ≤ .01), though major symptom group frequencies were similar (Table).

**Conclusion:**

During HIVE surveillance from October 2010 to December 2022, HPIVs were etiologic in 4.5% of ARIs. Symptoms were consistent across HPIVs, but healthcare-seeking, medication use, and coinfection frequencies differed significantly. Further study of HPIV ARI features in the community is needed as vaccine candidates enter late-stage trials.

**Disclosures:**

Arnold Monto, MD, Roche: Advisor/Consultant

